# Identification of small molecules that are synthetically lethal upon knockout of the RNA ligase Rlig1 in human cells†

**DOI:** 10.1039/d4cb00125g

**Published:** 2024-07-17

**Authors:** Florian M. Stumpf, Silke Müller, Andreas Marx

**Affiliations:** a Department of Chemistry, University of Konstanz Universitätsstraße 10 78457 Konstanz Germany andreas.marx@uni-konstanz.de; b Konstanz Research School Chemical Biology, University of Konstanz Universitätsstraße 10 78457 Konstanz Germany; c Department of Biology, University of Konstanz Universitätsstraße 10 78457 Konstanz Germany; d Screening Center, University of Konstanz Universitätsstraße 10 78457 Konstanz Germany

## Abstract

Rlig1 is the first RNA ligase identified in humans utilising a classical 5′–3′ ligation mechanism. It is a conserved enzyme in all vertebrates and is mutated in various cancers. During our initial research on Rlig1, we observed that Rlig1-knockout (KO) HEK293 cells are more sensitive to the stress induced by menadione than their WT counterpart, representing a type of chemical synthetic lethality. To gain further insight into the biological pathways in which Rlig1 may be involved, we aimed at identifying new synthetically lethal small molecules. To this end, we conducted a high-throughput screening with a compound library comprising over 13 000 bioactive small molecules. This approach led to the identification of compounds that exhibited synthetic lethality in combination with Rlig1-KO. In addition to the aforementioned novel compounds that diverge structurally from menadione, we also tested multiple small molecules containing a naphthoquinone scaffold.

## Abstract 2

Six unique compounds were found to be synthetically lethal in combination with Rlig1-KO in HEK293 cells. In addition, several naphthoquinones, which are similar to menadione, were evaluated in this context.

## Graphical abstract

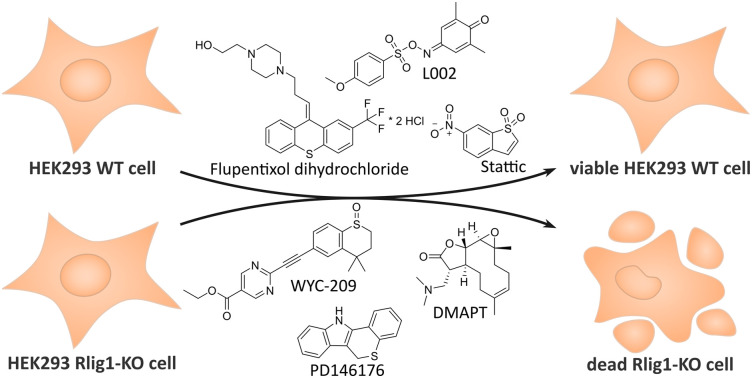

## Introduction

**Fig. 1:**
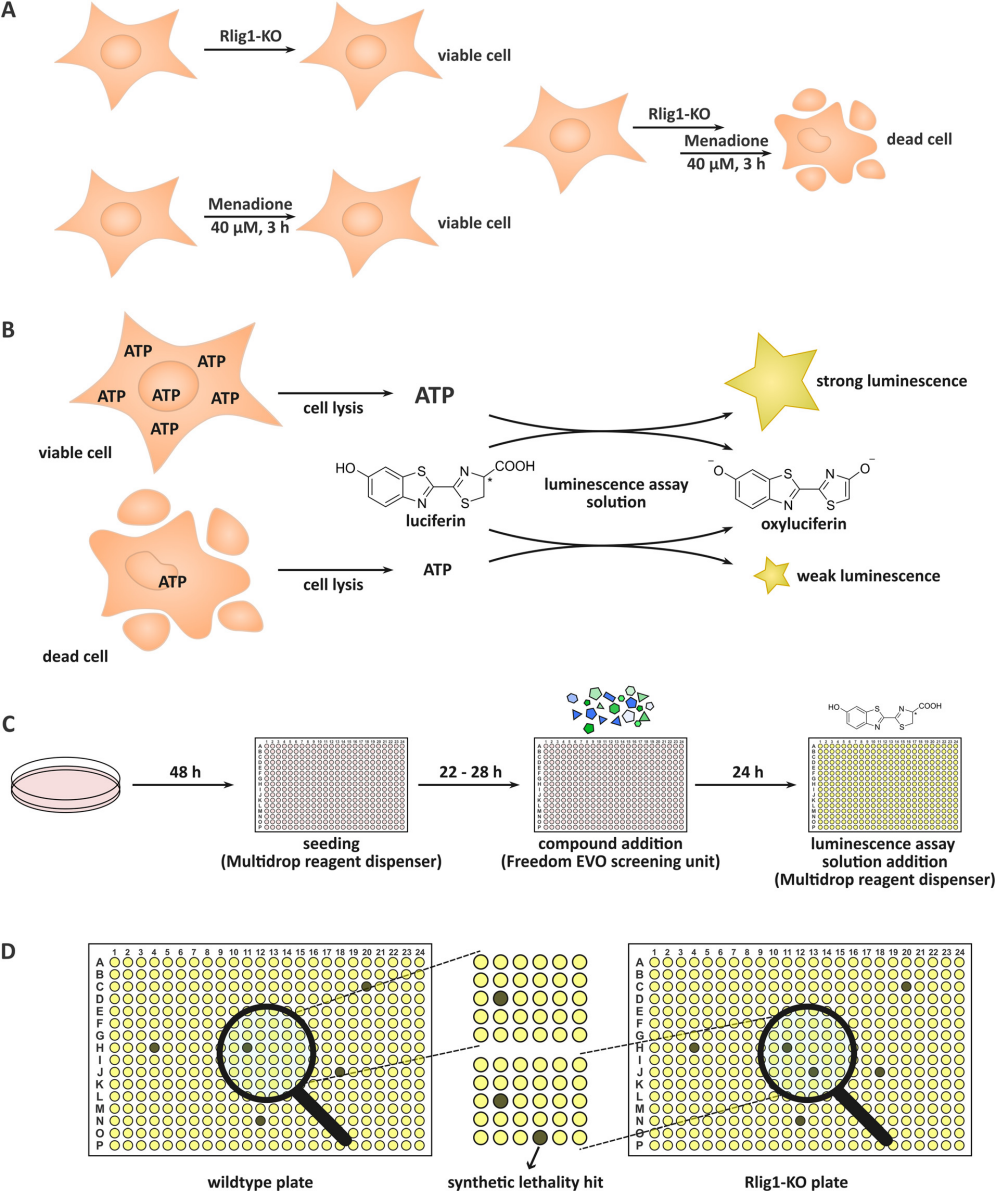
(A) While Rlig1-knock-out or menadione treatment alone does not lead to cell death, menadione is synthetically lethal in combination with the KO of Rlig1. (B) General principle of the commercially available luminescence assay. Viable cells contain more ATP than dead cells leading to higher luminescence output after lysis of the cells and reaction with the included luciferase. (C) Principle of the conducted initial screening. The cells are cultured in cell culture dishes. For the experiment, they are seeded in 384 well plates utilising a multidrop reagent dispenser. After incubation for 22–28 h, the compounds are added automatically by a Freedom EVO screening robot. 24 h after compound addition, the resulting viabilities are visualized by adding the assay reagent, again with a multidrop dispenser, and read out of the resulting luminescence. (D) Example for the detection of a synthetic lethal hit in the initial screening. In the Rlig1-KO plate one well (and therefore one compound) is inducing cell death which is not observable in the WT plate.

Rlig1 was very recently discovered by our group as a human 5′–3′ RNA ligase.^1^ Until then, the only proteinaceous RNA ligase known to be present in human cells was HSPC117,^2,3^ a 3′–5′ tRNA ligase involved in tRNA splicing which uses GTP for successive auto- and RNA-GMPylation.^4,5^ Rlig1 is a member of the nucleotidyltransferase family and operates *via* a classical three-step mechanism preferentially utilizing ATP.^6^ Initially, Rlig1 auto-AMPylates on lysine K57 in its catalytic site. This is followed by the transfer of the AMP moiety on the 5′-PO_4_ end of an RNA, yielding an RNA-adenylate intermediate. Consequently, an RNA 3′-OH group undergoes a nucleophilic attack on the RNA-adenylate, liberating AMP and resulting in the ligated product. While 5′–3′ RNA ligases, such as T4 Rnl of bacteriophages have been known and extensively utilised in biological laboratories for a considerable period of time,^7,8^ Rlig1 is the first ligase of this type to be identified in humans and is conserved in chordates.^1^ Additionally, Rlig1 has been found to be mutated in various cancers.^9–12^ Prior investigation by us showed that Rlig1-deficient human embryonic kidney (HEK293) cells exhibited increased sensitivity to menadione treatment in comparison to their wild-type (WT) counterparts. This led to an earlier death of Rlig1-knockout (KO) cells, providing an example of chemical synthetic lethality (Fig. 1A). Further experiments demonstrated that while reactive oxygen species (ROS) levels were comparable in both cells, the 28S rRNA of Rlig1-KO cells was degraded in contrast to the 28S rRNA of WT cells hinting at a role of Rlig1 in RNA maintenance and repair.^1,13^ Menadione (vitamin K3) is a commonly used oxidative stressor in human cell culture.^14–16^ Like other naphthoquinones, menadione produces superoxide radicals through its redox cycling in the cellular environment. These radicals can be further dismutated to H_2_O_2_.^17^ Menadione causes, dependent on the concentration, both apoptosis and necrosis in cells.^15^ The exact mechanism for this lethality is not clear, as the reactions to menadione treatment are thought to be diverse besides its ROS generation. These include p53 induction,^15^ spike of intracellular calcium,^15,18^ activation of the mitochondrial permeability transition pore^16,18^ and activation of PARP which can trigger NAD depletion as well as cytochrome *C* and AIF release from mitochondria.^16^ Interestingly, menadione is also known to inhibit the activation of NF-κB p65 and NF-κB p50^19^ and to arrest the cell cycle in the G2/M phase.^20^ The objective of this study was to identify small molecules that, like menadione, induce earlier death in Rlig1-deficient HEK293 cells in contrast to their WT counterpart. In other words, we sought to identify small molecules that are synthetically lethal with Rlig1-KO. By identifying compounds that are known to play a role in distinct biological pathways, insights into the role of Rlig1 in cells might be gained. To this end, a compound library comprising 13’006 bioactive small molecules was subjected to a high-throughput screening. The compounds in this library have been extensively researched and are typically capable of penetrating the cell membrane. Furthermore, they have shown to effect cell phenotypes by regulating signalling pathways. For the screening, both WT and Rlig1-KO cells were prepared and grown in parallel. We treated the plates with the small molecules and assessed the viability of the cells after 24 h. To ensure optimal reproducibility, a commercially available assay was adapted. By adding the assay solution, the cells were lysed, and the cellular ATP was converted into a luminescence output which could be interpreted. Living cells, containing significantly more ATP, exhibit a considerably higher luminescence output than their dead counterparts which contain substantially less ATP (Fig. 1B).

## Results and discussion

Before we conducted the initial screening, we ensured the functionality and reproducibility of the screening in test-experiments. First, we adapted a commercially available assay to our needs and reduced the amount of required assay solution. For this, we titrated the amount of solution and increased the incubation time to reliably access the resulting luminescence in our wells with our utilised cell count (Fig. S1, ESI†). As multiple compound plates were consecutively tested in a day, we also ensured the robustness of the screening for an incubation time of the cells between 22 and 28 h after seeding (robust *z*-score > 0.5). Menadione was used as a control (50 μM, positive control) in the screening. Treatment only with DMSO was used as negative control and set to 100% viability for the normalization. Additionally, systematic edge effects were corrected during data analysis.

### High-throughput screening of the bioactive compound library

In the initial screening, the compound library comprising 13 006 biologically active compounds was screened in a 384-well format (HY-L001, MedChemExpress). The objective was to identify compounds that exhibit preferential cytotoxicity towards HEK293 Rlig1-KO cells relative to WT cells. In an automated fashion, the desired amounts of compounds were directly added to the wells, containing cells and medium. This resulted in a final concentration of the compounds of 11.0 ± 0.31 μM. Following incubation of the cells with the small molecules and the procedure of the assay, the luminescence of the HEK293 Rlig1-KO cell plates were compared to the HEK293 WT plates (Fig. 1C and D). Only hits that exhibited at least a 25% higher viability in WT plates than in Rlig1-KO plates were chosen for further evaluation. Additionally, hits were excluded from further analysis if the apparent viability in one of the two plates (WT or KO cells) exceeded 120%. The robust *z*-factor of the screening was 0.86 ± 0.07 for all screened compound plates. For all screened compounds, the raw data and the normalized and corrected data of the screening can be found in the ESI.† Additionally, the luminescence distribution of the plates in the screening can be found in the ESI† (Fig. S2 and S3).

By the initial screening, a total of 23 compounds were identified as potentially synthetically lethal in combination with the Rlig1-KO, meeting the aforementioned criteria (see ESI†). It should be noted that only one distinct timepoint and concentration could be evaluated in this experimental setup. Therefore, this screening was by design constrained to only identify compounds that invoke a bigger change in viability in Rlig1-KO cells than in WT cells when treated at a concentration of 11 μM for 24 h. Incubation for a different timespan or at other concentrations could potentially result in other synthetically lethal hits. Seventeen of the 23 initially identified compounds were selected for further evaluation based on their hit quality and their solubility in DMSO at the desired concentrations for a suitable dose–response curve (see ESI†). In our case, this required a minimal solubility of the compounds of 45 mM. Of the seventeen preliminary hits, two exhibited a naphthoquinone scaffold, which is also present in menadione (vitamin K4 and NSC95397). Encouraged by this, the compound library was searched for additional molecules that were close to this structure to gain further insight into the mechanism by which menadione acts as a synthetically lethal compound in combination with Rlig1-KO. Nine additional compounds with minor structural alterations to the menadione molecule were selected for further experiments.

### Validation of the single-point screening hits

**Fig. 2:**
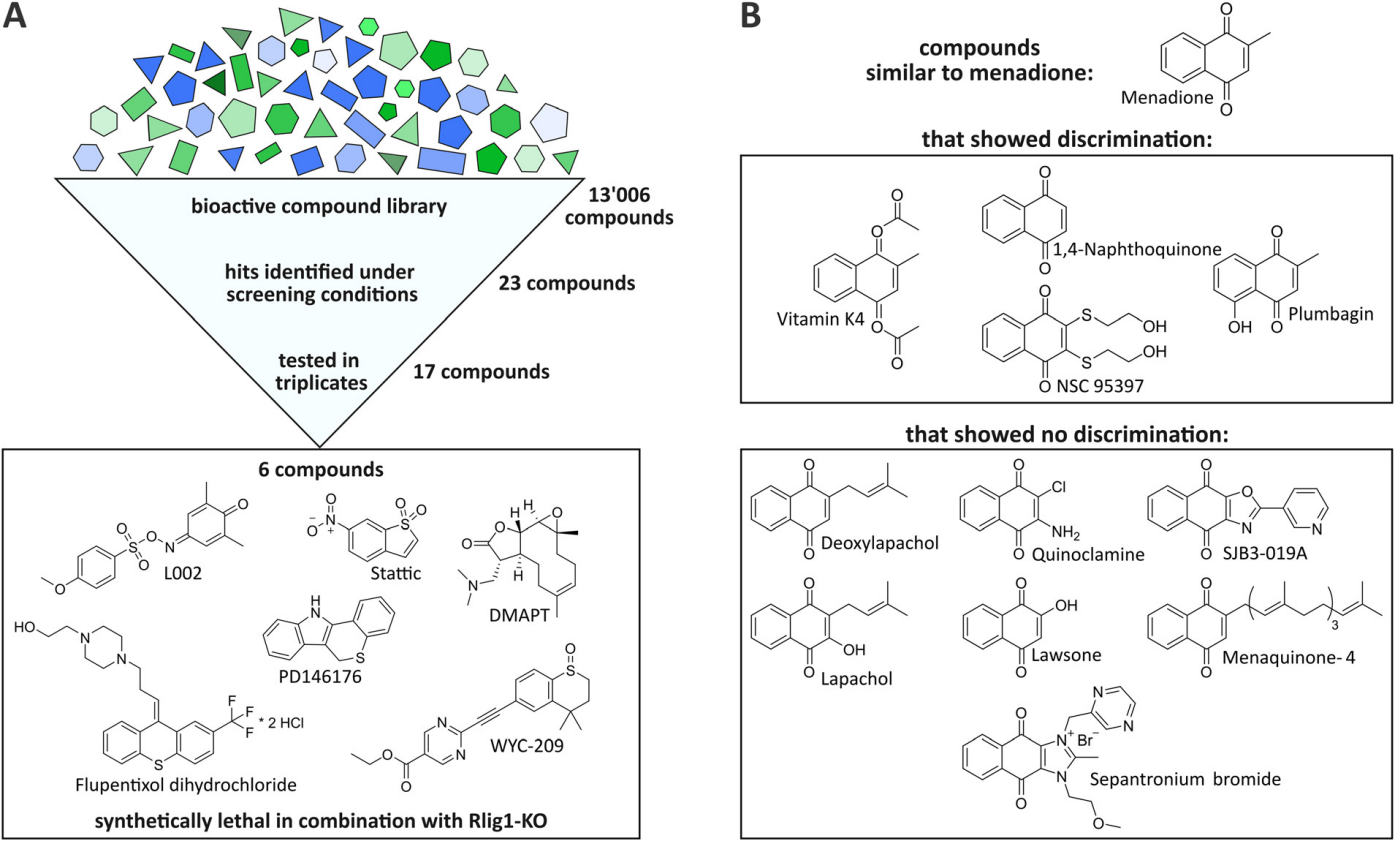
(A) After screening 13 006 bioactive compounds, 23 preliminary hits were detected. Of those, seventeen were tested in triplicates. Six compounds showed the tendency to be synthetically lethal in combination with Rlig1-KO in replicates. (B) A total of eleven compounds with naphthoquinone backbone (like menadione) were closer examined for synthetic lethality in combination with Rlig1-KO. Only four of them exhibited synthetic lethality in combination with Rlig1-KO in our experiments.

**Fig. 3:**
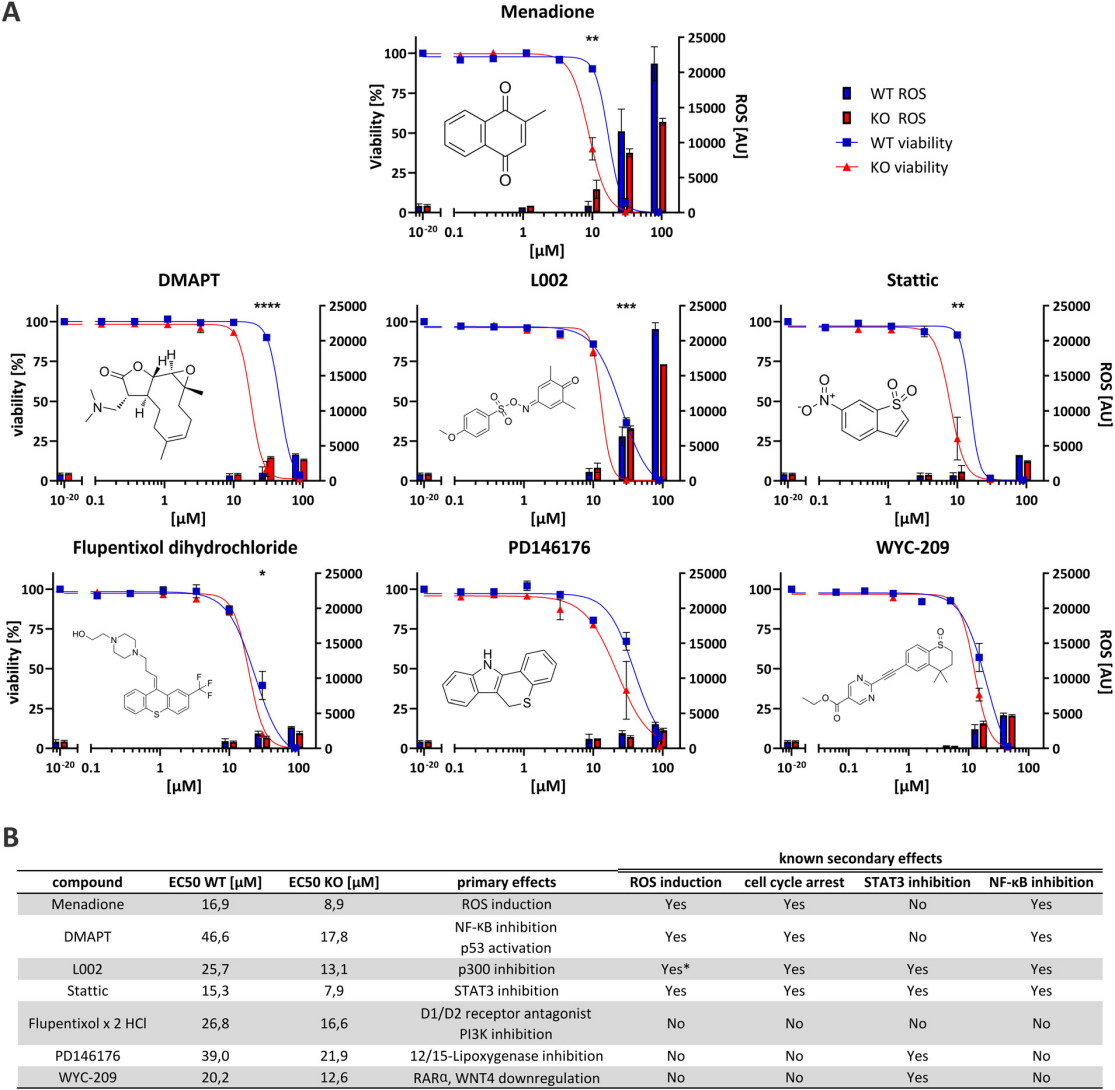
(A) Graphs of the six novel synthetically lethal compounds and of menadione as comparison. On the left *Y*-axis, the amount of viable cells are depicted with the corresponding EC50 curve (*n* = 3). The right *Y*-axis shows the relative amount of ROS (arbitrary units (AU)) (*n* = 2–3). Blue corresponds to the values for WT, red to the values for Rlig1 KO cells. Error bars are SEM. Stars indicate *p*-values (* < 0.05, ** < 0.01, *** < 0.001, **** < 0.0001) as determined by a two-tailed students *T*-test assuming equal variance. (B) Table listing of EC50 values and literature known primary effects of the compounds. Also listed are common literature known secondary effects. *While L002 is not literature known to induce ROS, we observed elevated ROS levels in our experiment similar to menadione.

The seventeen compounds identified as preliminary hits in the high-throughput screening were subjected to further examination in triplicates and in a dose-dependent manner in a 384-well format. The cells were cultivated in exactly the same way as in the initial screening. Of the seventeen, six structurally novel compounds and both naphthoquinone compounds showed a replicable tendency to alter the viability of HEK293 Rlig1-KO cells at lower concentrations than that of HEK293 WT cells. Consequently, these compounds were identified as synthetically lethal in combination with the Rlig1-KO (Fig. 2A). The EC50 of the six structurally new compounds were plotted and their literature known effects were compared (Fig. 3A and B). Four of the novel compounds (DMAPT, Stattic, L002 and flupentixol dihydrochloride) showed a significant difference in viability at one specific measured concentration.

DMAPT belongs to the family of parthenolides and is known to promote apoptosis in cancer cells by inhibiting the NF-κB complex and activating p53.^21,22^ The inhibition occurs by preventing IκB phosphorylation^23^ or by direct SH-alkylation in the NF-κB p65 subunit.^24^ The alkylation process is a result of the reactivity of parthenolides towards nucleophiles such as cysteine thiol-groups, in a Michael addition fashion.^25,26^ Stem-like cells exhibited elevated ROS levels, peaking 1 h after admission.^26–28^ Following this, depletion of GSH levels and disruption of calcium homeostasis,^25^ as well as dysfunction of mitochondrial activity and cytochrome *C* release occurred.^27,29^ Furthermore, DMAPT has been demonstrated to possess radiosensitising properties^30–32^ to induce cell cycle arrest at the G2/M phase^28,29^ and to inhibit DNA double-strand break repair.^30^

Stattic was identified as a STAT3 inhibitor.^33^ Like DMAPT, Stattic demonstrated to increase mitochondrial ROS formation in a dose-depending manner, resulting in mitochondria consuming less oxygen, less ATP production and a reduced calcium retention capacity.^34^ Furthermore, it has also been demonstrated that Stattic induces cell cycle arrest in the G2/M phase^35,36^ as well as inhibiting IκB phosphorylation which impedes NF-κB activation by preventing nuclear localization of NF-κB p65.^36^ Additionally, it has been shown to radiosensitize cancer cells.^35,37^

L002, a p300 inhibitor,^38,39^ PD146176, a 12/15-lipoxygenase inhibitor^40–42^ and WYC-209, an agonist of retinoic acid receptors (RARs)^43^ can also indirectly lead to STAT3 inhibition. The inhibition of p300 by L002 results in the blocking of acetylation of numerous proteins, including histones, p53, NF-κB p65 and STAT3.^38,44,45^ This then leads to the inhibition of STAT3 and NF-κB.^38,45^ L002 treated cells also showed growth arrest and apoptosis in cancer cells.^38^ PD146176 inhibits 12/15-lipoxygenase, thereby preventing the phosphorylation and activation of STAT3.^46^ WYC-209 promotes the binding of RARα to the WNT4 promoter, resulting in a reduction of WNT4 expression. Following WYC-209 treatment, the expression level of *p*-STAT3 was shown to be significantly impeded, mitigating STAT3 activation.^47^

Finally, flupentixol is a D1/D2 dopamine receptor antagonist^48,49^ and a PI3K inhibitor.^50^ While it has been used as a neuroleptic for many decades, new studies also suggest a use of it for the treatment of lung cancer.^50^

While the identified synthetically lethal compounds do not directly act on the same targets, the inactivation of STAT3 and NF-κB (especially p65) appears to be a common theme amongst them. It is noteworthy that menadione has also been demonstrated to inhibit NF-κB p65 activation in HEK293 cells.^19^ Furthermore, the arrest of the cell cycle, particularly in the G2/M phase, and increased ROS levels have been observed multiple times for the identified molecules in the literature, similar to menadione (Fig. 3B). As there are known cancers with Rlig1 mutations that erase the ligation activity of Rlig1,^1,9–12^ these compounds here might be able to kill these cancer cells while being without effect on healthy cells.

### Determination of reactive oxygen species in the confirmed synthetically lethal small molecules

Some of the identified compounds are known to increase ROS levels in cells, as evidenced in the literature. We wanted to understand if the synthetically lethal compounds exhibit a similar production of ROS in living cells like menadione in our experiments.^1^ Therefore, we adapted a commercially available assay for the quantification of ROS for our purpose (Fig. 3A). Intriguingly, only L002 exhibited ROS-levels comparable to those induced by menadione. This effect of L002 on human cells has not been previously described. It is noteworthy that both, DMAPT and Stattic, have been reported in the literature to induce certain types of ROS in cells. In our experiments, neither of the two compounds exhibited a significant change in ROS levels after 24 h. For DMAPT this effect was reported to be transient, with a peak of ROS observed after 1 h.^28^ Therefore, it is possible that our read-out of ROS after 24 h simply missed the right timeframe for detection. For Stattic, the effect was discovered under specific conditions when directly treating extracted rat mitochondria *in vitro*.^34^ This could be an explanation, why our experimental setup was not able to reproduce these results. The other three compounds showed no significantly elevated ROS levels, suggesting that different mechanisms are involved in the increased cell-death observed in Rlig1-KO cells.

### Menadione and structurally similar compounds

In addition to these novel hits, a total of eleven compounds exhibiting the naphthoquinone scaffold, which is also present in menadione, were investigated. Of these, four proved to be synthetically lethal in combination with Rlig1-KO in the examined concentration range (Fig. 2B and Fig. S4A and B, ESI†). It is noteworthy that, besides NSC-95397, these compounds are structurally very similar to menadione. Vitamin K4, a synthetic K vitamin, is most likely deacetylated to menadione in cellular context. Vitamin K2 (Menaquinone 4) did not demonstrate a different effect on the viability of Rlig1-KO cells in comparison to WT cells. Interestingly, vitamin K2 is also reported to not inhibit NF-κB in the same manner as menadione.^19^ In general, the compounds that did not differentiate between Rlig1-KO and WT cells all exhibited functional groups adjacent to the carboxy-groups of the naphthoquinone. It could be hypothesized that tolerated functional groups are very restricted at this side. In our experiments, longer apolar chains or electron-rich functional groups such as hydroxyl- or amine-groups were not tolerated.

## Conclusion

Rlig1 is a very recently discovered RNA ligase working in a manner not previously described in human cells.^1^ It is known that Rlig1 can covalently connect RNA strands in a classical three-step mechanism ligating a 5′-PO_4_ to a 3-OH. It was also reported that Rlig1 is binding to tRNAs *in vivo* and influences the tRNA levels in the brain of mice.^51^ Besides this, little is known about this enzyme and its potential intracellular functions and pathways. In our studies we saw the naphthoquinone menadione having a stronger toxic effect on Rlig1-deficient cells in contrast to WT cells. Menadione is a compound known to be able to invoke various cellular reactions and apoptotic pathways. To obtain new hints on which effect this synthetic lethality could be based, we decided to search for more small molecules which would discriminate Rlig1-KO and WT cells. In a single-point high-throughput screening of a bioactive compound library with more than 13 000 small molecules, we were able to identify 23 preliminary hits. Testing these further, we identified six compounds and two naphthoquinone compounds to be synthetically lethal in combination with Rlig1-KO. In addition to this, we tested nine further naphthoquinone-containing compounds of which two more were synthetically lethal. Intriguingly, many of the identified compounds share inhibitory effects of STAT3 and NF-κB. NF-κB inhibition is also reported for menadione. Additionally, elevated ROS levels and cell cycle arrest are reported or effects observed of some of them, similar to the effects of menadione. The identified compounds that are synthetically lethal in combination with Rlig1-KO might be able to kill cancer cells with Rlig1 mutations that abolish its RNA ligation activity. Additionally, they could provide a starting point for further research on the molecular and intracellular function of Rlig1 and help to further elucidate the role of this RNA ligase in RNA maintenance and repair.

## Author contributions

Conceptualization: FMS, AM; data curation: FMS, SM; formal analysis: FMS, SM; funding acquisition: AM; investigation: FMS, SM; project administration: AM; supervision: AM; visualization: FMS, SM; writing – original draft: FMS, AM; writing – review & editing: FMS, AM.

## Data availability

All data required is provided in the manuscript and the ESI.†

## Conflicts of interest

There are no conflicts to declare.

## Supplementary Material

CB-005-D4CB00125G-s001

CB-005-D4CB00125G-s002

CB-005-D4CB00125G-s003
