# Supplementary material for: Identification of small molecules that are synthetically lethal upon knockout of the RNA ligase Rlig1 in human cells
Source: RSC Chem Biol. 2024 Jul 17;5(9):833–40. doi: 10.1039/d4cb00125g (PMC11353076; doi:10.1039/d4cb00125g)
Supplement: CB-005-D4CB00125G-s003 [file CB-005-D4CB00125G-s003.pdf]

Electronic Supplementary Material (ESI) for RSC Chemical Biology.
This journal is © The Royal Society of Chemistry 2024

# Supporting Information

## General Methods

For optimal reproducibility in the screening, exclusively the following material was used throughout the initial screening:

- CellTiter-Glo® 2.0, Promega, G9242 Lot: 0000562820
- FCS, Sigma, S0615 Lot: 0001659225
- Dulbecco's Modified Eagle's Medium (DMEM) Glutamax, Thermo-Fisher Scientific, Ref: 61965-026 Lot: 2613622
- 384-well plates: Greiner, Ref: 781092 Lot: E23023EF

CRISPR/Cas9-mediated generation of a C12ORF29 knock-out HEK293 cell line was ordered by our group from trenzyme GmbH. The parental cells of the knock-out were used as the HEK293 WT cells.

The medium used for culturing the cells was DMEM Glutamax + 10% FCS. The cells were cultured in 15 cm standard cell culture plates (Sarstedt). The cells were passaged every other day, and the cells were incubated at 37 °C, 7.5% $CO_2$ and 95% humidity if not noted differently.

DPBS and TrypLE™ was obtained from Thermo-Fisher, DMSO (Cat. No. D2438) and menadione (Cat no. 47775) was obtained from Sigma-Aldrich. The CellTiter-Glo® 2.0 Cell Viability Assay and the ROS-Glo™ $H_2O_2$ Assay was obtained from Promega. The bioactive compound library (HY-L001) and single compounds for EC50 determination were obtained from MedChemExpress.

For cell counting, a Luna™ automated cell counter with Luna™ cell counting slides (L12001) was used.

Thermo-Scientific™ Multidrop™ Combi was used in combination with cassettes from Steinle Labortechnik for cell seeding (standard tube dispensing cassette) and Thermo-Scientific (small tube metal tip dispensing cassete, Cat no. 24073295) for CellTiter-Glo® 2.0 solution addition.

For the compound transfer on the 384 well plates, a Tecan freedom evo screening robot was used.

384 well plates were incubated on an Eppendorf ThermoMixer C with a plate adapter and ThermoTop.

For the luminescence read-out, a Tecan Spark was used in combination with Magellan software (25°C, 800 ms settle time, 250 ms integration time, output = counts/s).

For multipipetting, Integra Viaflo or Integra Voyager pipettes were used. For automated multipipetting an Integra assist plus pipetting robot was used.

**Screening procedure**

Two days before the screening experiment 8 x $10^6$ cells in 20 ml medium were seeded in 15 cm cell culture dishes. On the day of the screening experiment, the medium was removed and 3 ml TrypLE™ was added to the cells, distributed evenly, and the cells were incubated for 5 min. 12 ml medium was added, and the cells were resuspended. The cell suspension was further diluted by adding 30 ml medium per plate. Afterwards, the cells were counted twice and the counts were averaged. Medium was added to result in a cell solution containing 2.5 x $10^5$ cells/ml. 40 µl of the cell suspension was seeded per well (10'000 cells per well) in 384 well plates with a multidrop (Steinle Labortechnik cassette, medium speed, whole plate, 40 µl per well, 40 µl pre-dispense). The cells were incubated for 22-26 h before the compound transfer was conducted.

After the incubation time, the compound transfer from the bioactive compound library was conducted with the Tecan freedom Evo screening robot. To row 23 and 24 only the carrier DMSO without compounds was added. Afterwards, 20 µl medium was added to all rows of the plate except for row 24 to evenly distribute the compounds in the well (Steinle Labortechnik cassette, high speed, row 1-23, 20 µl per well, 20 µl pre-dispense). The final concentration of compounds in the wells was determined to be 11 µM. To row 24, 20 µl of 0.15 mM menadione in medium was added as a killing control per hand with a multipipette (end concentration = 50 µM). Row 23 was used as negative control (later set to 100% viability) and row 24 was used as positive control. The plate was incubated for 24 h.

After the incubation time, 15 µl of CellTiter-Glo® 2.0 solution was added to each well with a multidrop (Thermo-Scientific cassette, medium speed, whole plate, 15 µl per well, 20 µl pre-dispense). The plate was centrifuged (800 g, 1 min) and incubated (25°C, 800 rpm, 40 min). Directly afterwards, the luminescence was read out with a Tecan Spark and the data analysis of the screening was done with KNIME. Here the robust z-factor was calculated using the negative control row (row 23) and the positive control row (row 24). Additionally, the data was normalized (negative control (row 23) was set to 100% viability) and edge effects were corrected. Then, after screening WT and KO cells with the same compound plates, the data was sorted, filtered and matched to identify the hits.

**Determination of EC50**

The compounds that were determined as preliminary hits in the screening were ordered from MedChemExpress. The compounds were dissolved in DMSO to result in a concentration of 45 mM (22.5 mM if they were not soluble at higher concentrations). Afterwards a serial dilution was performed *via* automated multipipetting (resulting in 7 different concentrations, each concentration 1/3 diluted from the concentration before). The resulting compound plates in DMSO were stored at -20°C until further use.

The cells were cultivated and seeded for the experiment in 40 µl volume in 384 well plates in the exact same way as described in the screening procedure above.

At the day of the compound transfer, the compound plates containing the diluted compounds in DMSO were equilibrated to room temperature on the ThermoMixer right before the experiment (40 min, 25°C, 400 rpm). After a short centrifugation (1 min, 800 g), 1.5 µl of the compounds were given to 250 µl medium *via* automated multipipetting. The resulting compound plate in medium was then used for the compound transfer. For this, 20 µl were given to the grown cells *via* automated multipipetting and the cells were incubated for 24 h. The final concentration of DMSO in the cell medium amounted to 0.2 %.

CellTiter-Glo® 2.0 solution addition and luminescence read-out were conducted in the same way as in the screening procedure above. For each replicate four technical replicates were measured and averaged. Values with over 150% luminescence of the negative control were excluded. Graphs and curves were plotted with Graphpad Prism Version 10.2.3. For the EC50 curves a non-linear regression (log(inhibitor) vs response – variable slope) was used. The bottom was forced to 0 for curves that did not reach a stable baseline in the regression.

**Determination of ROS**

The cells were cultivated and seeded for the experiment in 40 µl volume in 384 well plates in the exact same way as described in the screening procedure above.

The compound transfer was done manually with a multipipette from a compound plate in medium (prepared as described above in the EC50 determination section). For this, 20 µl of the compound in medium with the desired concentration was added to the cells and the cells were incubated for 21 h. Afterwards, the plate was gently shaken (5 min, 37°C, 300 rpm) and 40 µl of the present medium was removed *via* automated multipipetting without disturbing the cells. Then, 5 µl $H_2O_2$ substrate from the ROS-Glo™ $H_2O_2$ assay (prepared as described in the manufacturer's instructions) was added to each well and the plate was centrifuged (1 min, 300 g) and gently shaken (5 min, 37°C, 300 rpm). The cells were incubated for 3 h. After 3 h (in total 24 h after compound addition), 25 µl of the Luciferin detection reagent with D-Cysteine and signal enhancer solution (prepared as described in the manufacturer's instructions) was added per well. The plate was centrifuged (1 min, 800 g) and incubated (40 min, 25 °C, 800 rpm). The luminescence read-out was conducted in the same way as in the screening procedure above. The raw data was plotted in GraphPad Prism Version 10.2.3.

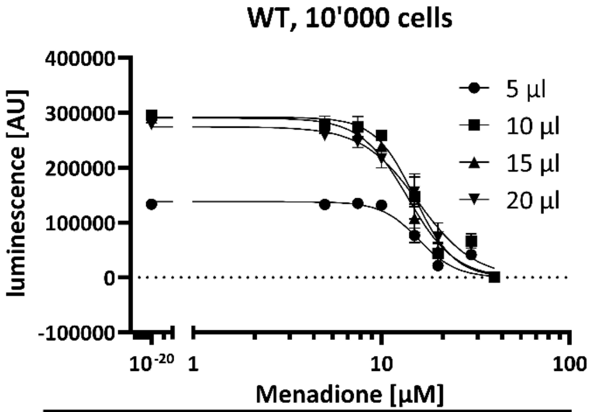


| | 5 µl | 10 µl | 15 µl | 20 µl |
|---|---|---|---|---|
| Bottom | 0 | 0 | 0 | 0 |
| Top | 138040 | 291891 | 290603 | 274422 |
| HillSlope | -4.39 | -4.11 | -3.47 | -2.93 |
| EC50 | 15.82 | 15.02 | 13.83 | 15.72 |

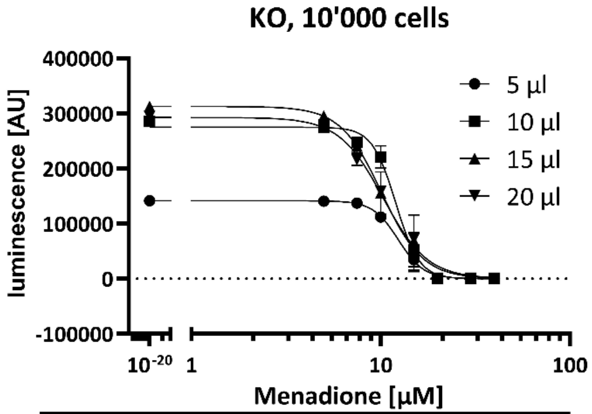


| | 5 µl | 10 µl | 15 µl | 20 µl |
|---|---|---|---|---|
| Bottom | 0 | 0 | 0 | 0 |
| Top | 141438 | 274814 | 312547 | 292514 |
| HillSlope | -6.45 | -7.01 | -3.98 | -3.71 |
| EC50 | 12.41 | 12.03 | 10.12 | 10.33 |

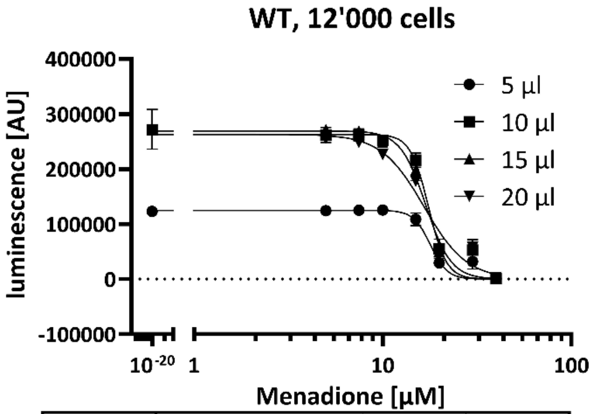


| | 5 µl | 10 µl | 15 µl | 20 µl |
|---|---|---|---|---|
| Bottom | 0 | 0 | 0 | 0 |
| Top | 124800 | 262612 | 268906 | 262940 |
| HillSlope | -9.78 | -9.16 | -6.51 | -3.85 |
| EC50 | 17.92 | 17.53 | 16.96 | 16.60 |

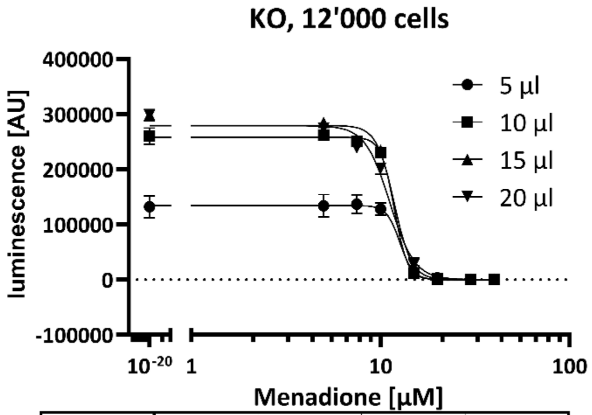


| | 5 µl | 10 µl | 15 µl | 20 µl |
|---|---|---|---|---|
| Bottom | 0 | 0 | 0 | 0 |
| Top | 134328 | 258056 | 279438 | 279249 |
| HillSlope | -12.51 | -12.39 | -9.39 | -6.68 |
| EC50 | 12.68 | 11.83 | 11.81 | 11.22 |

Fig. S1. Titrating of the CellTiter-Glo© 2.0 solution to determine the required amount for reliable luminescence output. The cells were seeded and treated with menadione in the same way as in the determination of the EC50. In both cell counts, 10’000 and 12’000 cells, only 5 µl solution led to a visible decreased luminescence output. Between 10 µl, 15 µl and 20 µl no significant difference could be observed. For the initial screening, 10’000 cells and 15 µl solution was used.

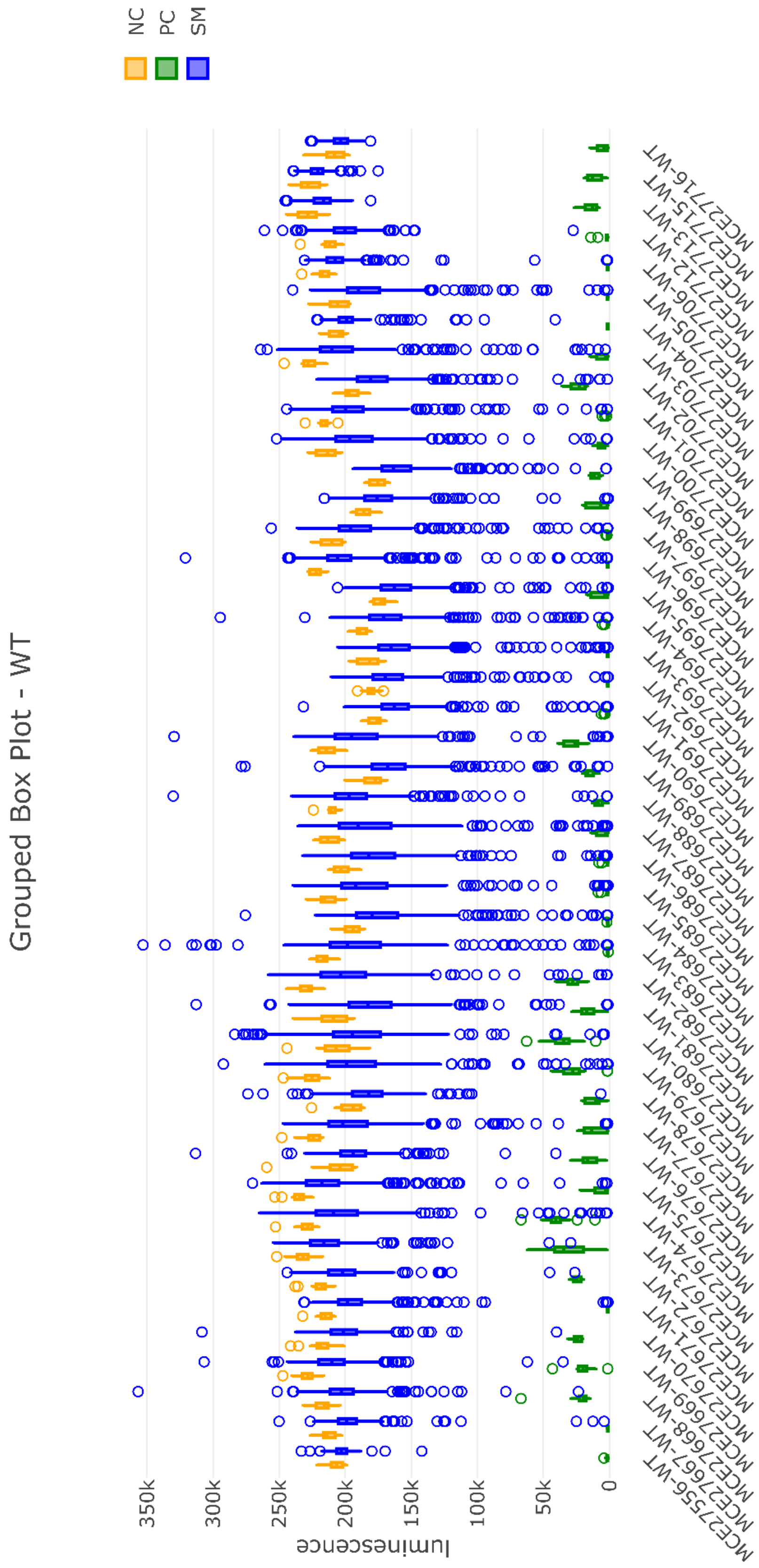


Fig. S2 Luminescence distribution of the initial screening of the HEK293 WT cell plates. The luminescence of the samples is depicted in blue. The negative controls (only DMSO carrier) are depicted in yellow and the positive controls (50 µM menadione) are depicted in green.

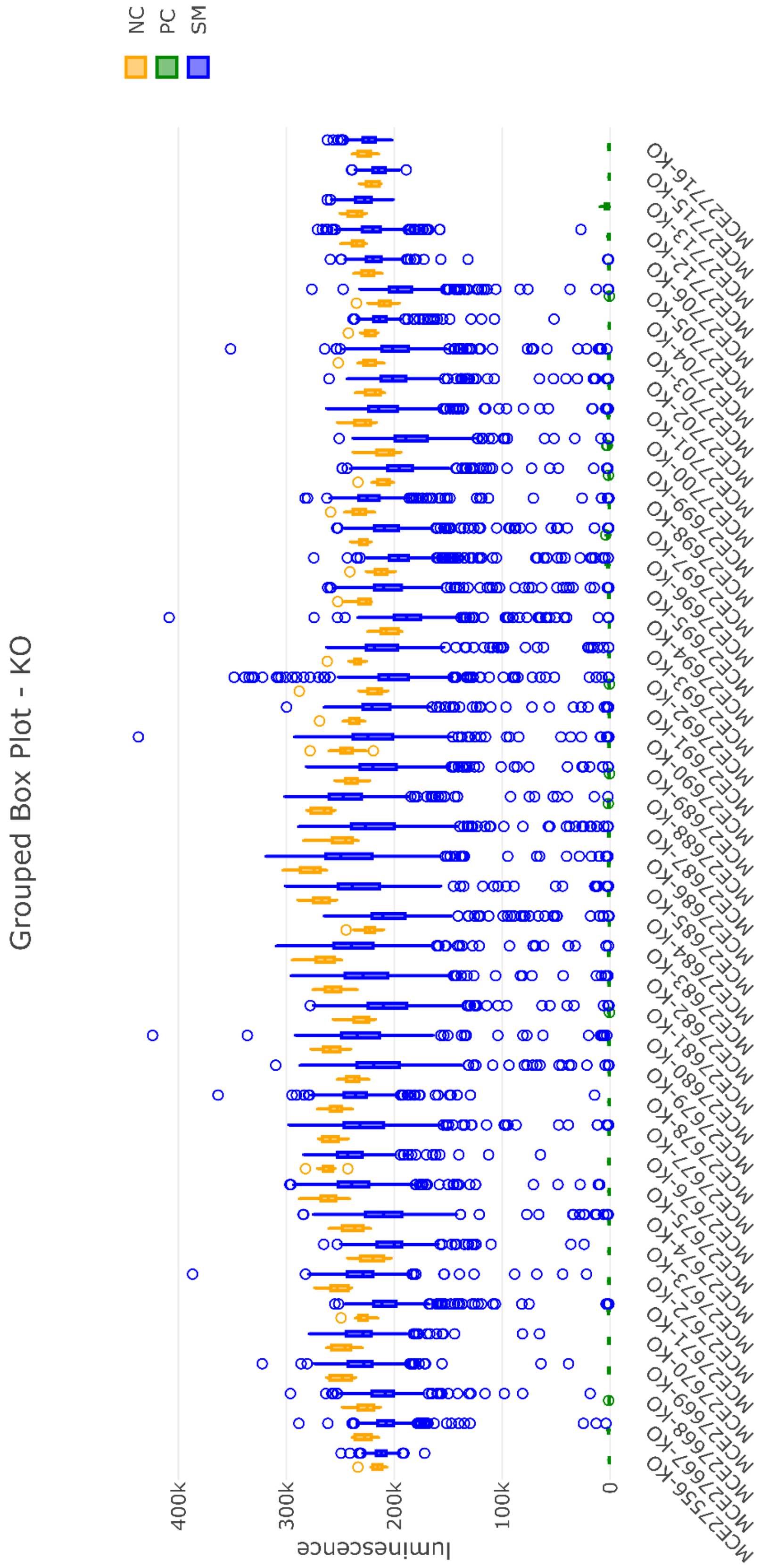


Fig. S3 Luminescence distribution of the initial screening of the HEK293 Rlig1-KO cell plates. The luminescence of the samples is depicted in blue. The negative controls (only DMSO carrier) are depicted in yellow and the positive controls (50 µM menadione) are depicted in green.

**A**

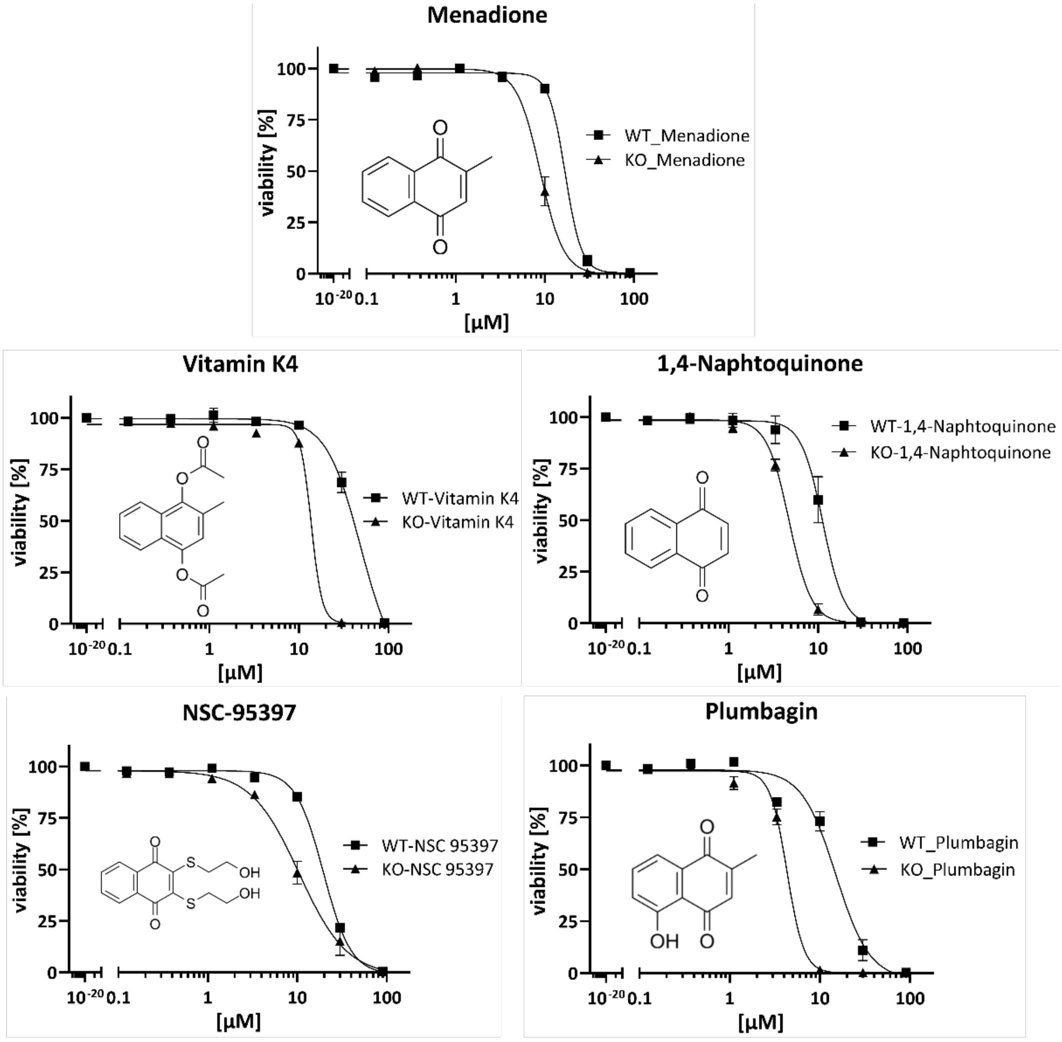


**B**

| compound | EC50 WT [µM] | EC50 KO [µM] |
|---|---|---|
| Menadione | 16,9 | 8,9 |
| Vitamin K4 | 48,7 | 13,7 |
| 1,4-Naphthoquinone | 11,3 | 4,8 |
| NSC-95397 | 19,6 | 10,5 |
| Plumbagin | 15,0 | 4,3 |

Fig. S4 (A) EC50 curves of the synthetically lethal naphthoquinone compounds. On the Y-axis, the share of viable cells is depicted with the corresponding EC50 curve (n=3). Error bars are SEM. (B) EC50 values for the WT and the KO cells when treated with the respective compounds (24 h).
